# Improving cellulase production by *Aspergillus niger* using adaptive evolution

**DOI:** 10.1007/s10529-016-2060-0

**Published:** 2016-02-15

**Authors:** Aleksandrina Patyshakuliyeva, Mark Arentshorst, Iris E. Allijn, Arthur F. J. Ram, Ronald P. de Vries, Isabelle Benoit Gelber

**Affiliations:** Fungal Molecular Physiology, CBS-KNAW Fungal Biodiversity Centre, Utrecht University, Utrecht, The Netherlands; Molecular Microbiology and Biotechnology, Leiden University, Leiden, The Netherlands

**Keywords:** *Aspergillus niger*, Adaptive evolution, Cellulose, NADPH oxidase, NoxR

## Abstract

**Objectives:**

To evaluate the potential of adaptive evolution as a tool in generating strains with an improved production of plant biomass degrading enzymes.

**Results:**

An *Aspergillus niger* cellulase mutant was obtained by adaptive evolution. Physiological properties of this mutant revealed a five times higher cellulose production than the parental strain. Transcriptomic analysis revealed that the expression of *noxR,* encoding the regulatory subunit of the NADPH oxidase complex, was reduced in the mutant compared to the parental strain. Subsequent analysis of a *noxR* knockout strain showed the same phenotypic effect as observed for the evolution mutant, confirming the role of NoxR in cellulose degradation.

**Conclusions:**

Adaptive evolution is an efficient approach to modify a strain and activate genes involved in polysaccharide degradation.

## Introduction

Cellulose is the most abundant carbohydrate in nature. It is a linear polymer of d-glucose residues linked by β-1,4-glucosidic bonds and forms major structural plant cell wall component. Due to its partially crystalline structure, it is more recalcitrant to enzymatic degradation than other plant cell wall polysaccharides. The cellulose-degrading system in fungi includes three classes of hydrolytic enzymes: β-1,4-endoglucanases (EGL) and exoglucanases/cellobiohydrolases (CBH) that hydrolyze cellulose into gluco-oligosaccharides which are then further degraded into d-glucose by the action of β-glucosidases (BGL) (van den Brink and de Vries 2011). A fourth class of oxidative enzymes has been recently described: the lytic polysaccharide mono-oxygenases (Morgenstern et al. 2014). Cellulases have wide range of applications including production of chemicals, fuel, food, brewery and wine, animal feed, textile and laundry, and pulp and paper. However, from a commercial point of view, the cost of cellulose-degrading enzymes is a major barrier to the economical production of biochemicals and second generation biofuels. Therefore, production and optimization of cellulases from a wide range of fungi have been studied (Lynd et al. 2002). The industrial filamentous fungus, *Trichoderma reesei*, has been extensively studied for the production of cellulase preparations. It produces high levels of endoglucanases and cellobiohydrolases but low levels of β-glucosidases which are essential for full hydrolysis of cellulose (Singhania et al. 2013). *Aspergillus niger* is also a well-known, genetically tractable and industrial fungus that proficiently degrades plant cell wall polysaccharides. Its genome contains a complete set of cellulolytic genes but *A. niger* grows poorly on cellulose (Coutinho et al. 2009). During growth on plant biomass *A. niger* secretes high BGL and low CBH and EGL levels compared to *T. reesei* (Hanif et al. 2004).

One of the outstanding features of microorganisms is their capability to adapt rapidly to various environmental conditions. This ability has been applied to improve yeast strains for different biotechnological applications by conducting adaptive laboratory evolution (Bachmann et al. 2015; Kutyna et al. 2012). *Aspergillus nidulans* was exposed to adaptive evolution, resulting in phenotype changes as well as a higher fitness in the adapted population (Schoustra et al. 2009; Schoustra and Punzalan 2012). However, this evolutionary approach has not yet been applied to generate fungal strains with enhanced enzyme production. We describe here the generation and characterization of a stable evolved isolate, which showed improved cellulase production.

## Methods

### Strains, media and growth conditions

All cultures were performed at 30 °C. The *A. niger* strains, listed in Table 1, were grown on malt extract/agar (MEA) to produce spores, which were harvested using ACES buffer [10 mM N-(2-acetamido)-2-aminoethanesulfonic acid (Sigma-Aldrich), 0.02 % Tween 80, pH 6.1–7.5]. For liquid cultures, 50 ml of aspergillus minimal medium (MM) (de Vries et al. 2004) containing 1 % (w/v) α-cellulose (Sigma-Aldrich) in a 250 ml flask was inoculated with 10^6^ spores ml^−1^ and incubated at 30 °C, 250 rpm.

**Table 1:** List of *A. niger* strains

Strain	Genotype	Description	Reference
N402	*cspA1*	Parental strain	(Bos et al. 1988)
CBS 140717	*cspA1*	Cellulose adaptive evolved strain	This study
MA78.6	Δ*kusA*::*amdS*	Reference Δ*noxR* (ref_ Δ*noxR*)	(Carvalho et al. 2010)
MA75.2	Δ*kusA*::*amdS*; Δ*noxR*::*AopyrG*	Δ*noxR*	(Kwon et al. 2011)
MA 82.2	Δ*kusA*::*amdS*; Δ*noxA*::*AopyrG*	Δ*noxA*	(Kwon et al. 2011)

For agar plates, 1.5 % agar was added to MM containing 1 % (w/v) α-cellulose or 1 % (w/v) α-cellulose and 1 % (w/v) glucose. Agar plates were inoculated in the center with 2 µl spore suspension (10^6^ ml^−1^) and incubated at 30 °C.

### Adaptive evolution experiment

*A. niger* N402 was inoculated with 5 μl spore suspension at (10^6^ ml^−1^) in a Petri dish containing MM with 1 % (w/v) α-cellulose and 1.5 % (w/v) agar. We propagated this lineage by serial transfers every 5 days for 24 weeks. Each generation, the complete spore plate was harvested, the spore suspension mixed extensively and 5 μl was plated on fresh medium. Two evolving lineages were conducted in parallel. The fitness of the colonies gradually increased until a good growth on α-cellulose was observed. Several colonies were purified and the colony showing the best growth on α-cellulose was selected for further studies. The resulting improved strain was deposited at the CBS collection, referred as CBS 140717.

### RNA isolation and microarray analysis

Liquid cultures of *A. niger* N402 and CBS 140717 were pre-grown for 16 h in MM with 2 % (w/v) fructose after which mycelial samples were transferred to MM with 25 mM xylose and incubated for 2 h. The mycelium was harvested and used for RNA isolation. The concentration of RNA was measured at A_260_, while the quality was analyzed with an Agilent 2100 bioanalyzer, using an RNA6000 LabChip kit (Agilent Technology, Palo Alto, CA, USA). Microarray analysis was performed as previously described (Levin et al. 2007).

### Enzyme assays and protein profiles

Culture samples (1.5 ml) from 1 % α-cellulose shake-cultures were taken each day for 10 days and centrifuged for 10 min, at ~10,000×*g*, 4 °C to separate the solid fraction from the supernatant. The filtrates from duplicate flask cultures were assayed using *p*-nitrophenol-linked substrates (4-nitrophenyl β-D-xylopyranoside, 4-nitrophenyl β-D-cellobioside, 4-nitrophenyl β-D-glucopyranoside; Sigma-Aldrich). The assays contained in 100 μl, 5-40 μl sample, 10 μl 0.01 % *p*-nitrophenol-linked substrates, 0-35 μl Mili-Q water and 50 mM sodium acetate buffer (pH 5.0). Samples were incubated in microtiter plates for 2 h at 30 °C. Reactions were stopped by addition of 100 μl 0.25 M Na_2_CO_3_. Absorbance was measured at 405 nm in a microtiter plate reader. The activities were calculated using a standard curve ranging from 0 to 20 nM *p*-nitrophenol per assay volume.

Culture filtrates, 20 µl, taken at day 10 of cultivation were loaded onto 12 % (w/v) SDS-PAGE gels. The gel was silver stained. Total protein concentration was measured by BCA method (Pierce BCA Protein Assay Kit, ThermoFisher).

## Results and discussion

### Adaptive evolution of the strain

**Fig. 1:**
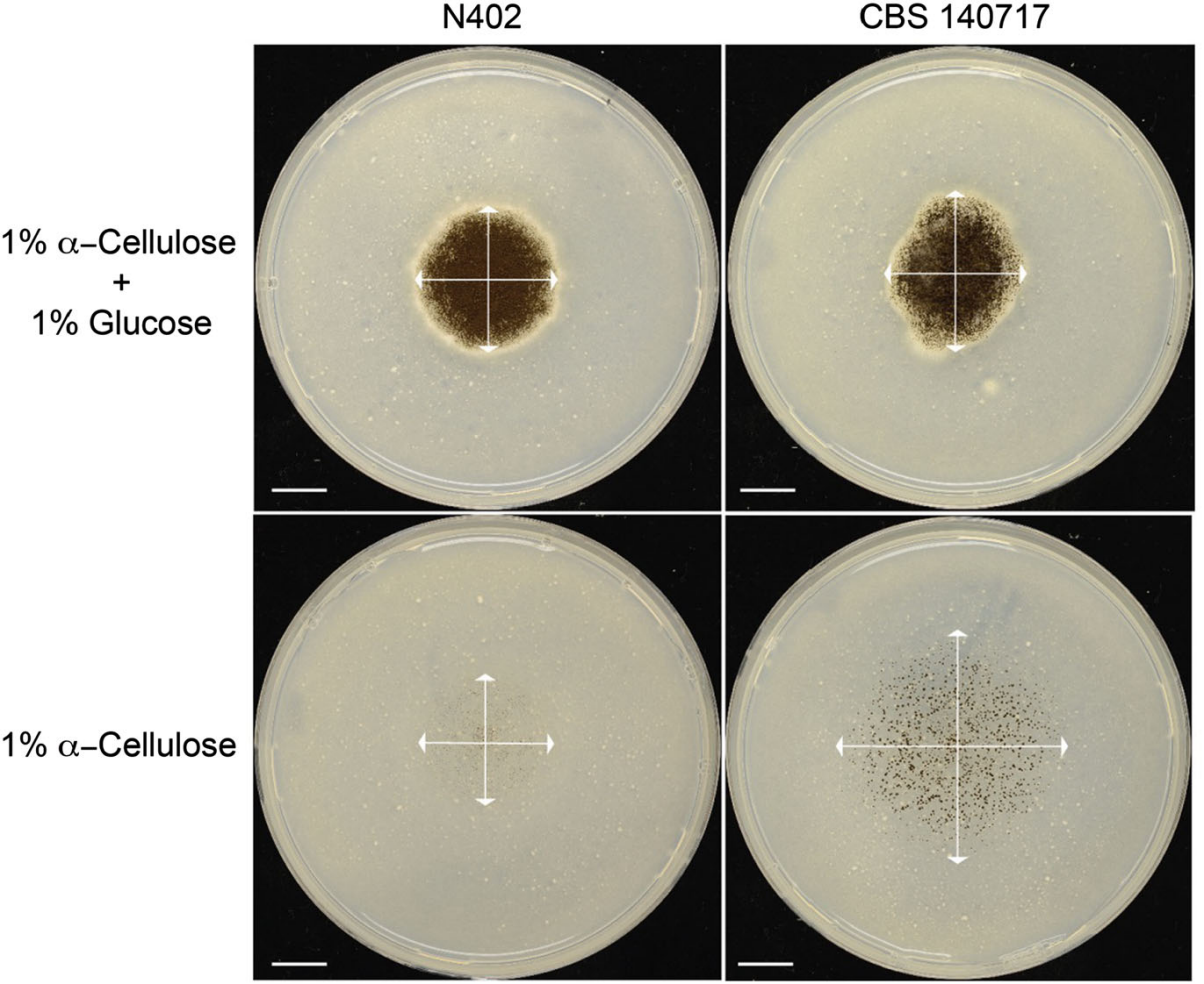
*A. niger* cellulose evolved strain CBS 140717 and parental strain N402 grown on α-cellulose and glucose + α-cellulose

In order to evaluate the potential of adaptive evolution in filamentous fungi, we chose to apply this approach on *A. niger* N402 and select for strains with improved cellulase activity (see Materials and methods). The poor growth of *A. niger* N402 on cellulose suggested this substrate as a good substrate for selective mutations caused by adaptive evolution. Several colonies were purified and the colony showing the best growth on α-cellulose was selected for further studies. The selected evolved cellulose mutant, CBS 140717, showed similar growth to N402 on cellulose in the presence of glucose but a clear improved growth was visible on cellulose as sole carbon source (Fig. 1).

### Gene expression profile of the parental and evolved strains

Cellulolytic genes in *A. niger* are under control of the (hemi-)cellulolytic regulator XlnR (van Peij et al. 1998) that responds to the presence of xylose. Therefore, to obtain insight in the factor causing the improved cellulase production, a single sample microarray was performed on this mutant grown in xylose and compared to the parental strain grown in the same conditions. Overall, no significant increase in cellulolytic gene expression was observed. Induction for 2 h with xylose may have been too short to see an immediate effect on cellulolytic gene expression. Interestingly, the expression level of the *A. niger noxR* homolog has strongly decreased in the CBS 140717 strain, from a signal value of around 50–1.5. In *Podospora anserina* deletion of this gene (*PaNoxR*) resulted in reduced cellulase production (Brun et al. 2009). Thus, we hypothesized a similar role in the regulation of cellulase production in *A. niger*.

### Characterization of the *A. nigernoxR* knockout strain

To confirm the role of *noxR* in cellulase production in *A. niger*, we compared CBS 140717 and its parental strain to the knockout mutant *noxR* (Kwon et al. 2011) and its parental strain.

**Fig. 2:**
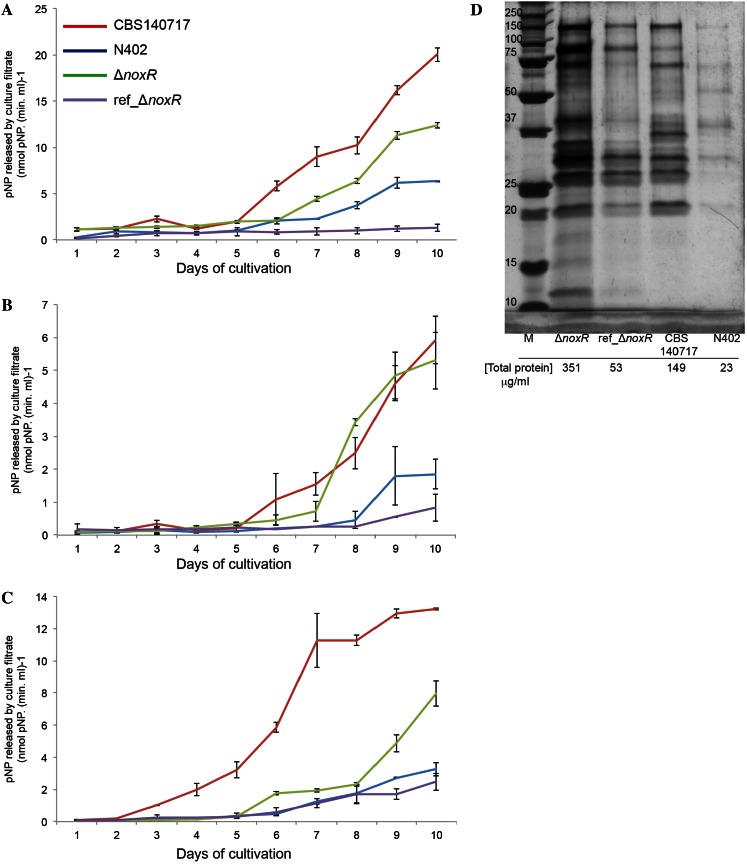
β-1,4-D-Glucosidase (**a**), cellobiohydrolase (**b**) and β- xylosidase (**c**) activities of culture filtrates produced by *A. niger* strains grown in cellulose shaken cultures. Extracellular protein profile on SDS-PAGE and total protein concentration (**d**) of CBS 140717, N402, Δ*noxR* and reference strain (ref_ Δ*noxR*) of *A. niger*

BXL, CBH and BGL activities were measured during 10 days of cultivation, demonstrating increase of these activities over time (Fig. 2a–c). All three enzyme activities showed higher expression for CBS 140717 and Δ*noxR* strains compared to their reference strains.

The SDS-PAGE patterns of CBS 140717and N402 show distinct differences in terms of absence and altered intensity of protein bands (Fig. 2d). A similar pattern was observed when comparing Δ*noxR* and ref_Δ*noxR* strains with the absence of some proteins or reduced intensity of protein bands in ref_Δ*noxR* (Fig. 2d). Higher secreted protein concentrations (6-7-fold) were measured for both CBS 140717 and Δ*noxR* compared to N402 and ref_Δ*noxR* strains, respectively. As NoxR interacts with NoxA in *A.niger* (Kwon et al. 2011), we also tested the Δ*noxA* strain (data not shown), but this strain was comparable to the reference strain.

Our adaptive evolution approach was efficient to modify *A. niger* N402 in a milder way than the classical random mutagenesis (e.g. UV), which was for instance used to obtain the *T. reesei* RUT-C30 cellulose producer (Montenecourt and Eveleigh 1977). Advantageously, this method is likely to induce less random mutations because mutations are linked to the selection for the improved fitness of the strain. These results demonstrate the value of adaptive evolution as a tool to generate strains with improved enzyme production. In addition, these strains can be used to identify novel factors that affect enzyme production, such as NoxR. In future studies, we aim to address the molecular mechanism of NoxR in this process.

**In conclusion**, the evolved strain, CBS 140717, showed an increase of about five-fold in cellulase production compared to *A. niger* N402 and analysis of this strain revealed a role for NoxR (RiaA) in cellulase production in *A. niger*.
